# Supplementary material for: Pyruvate Dehydrogenase Kinase 1 inhibition mediated oxidative phosphorylation enhancement in cartilage promotes osteoarthritis progression
Source: BMC Musculoskelet Disord. 2023 Jul 20;24:597. doi: 10.1186/s12891-023-06585-6 (PMC10357736; doi:10.1186/s12891-023-06585-6)
Supplement: Supplementary file 1 — Additional file 1. [file 12891_2023_6585_MOESM1_ESM.zip › KEGG.Gsea.1653622432464/KEGG_O_GLYCAN_BIOSYNTHESIS.html]

Details for gene set KEGG\_O\_GLYCAN\_BIOSYNTHESIS[GSEA]

|  || Dataset | 55457\_collapsed\_to\_symbols.cls.cls #OA\_versus\_normal.cls.cls #OA\_versus\_normal\_repos |
| Phenotype | cls.cls#OA\_versus\_normal\_repos |
| Upregulated in class | OA |
| GeneSet | KEGG\_O\_GLYCAN\_BIOSYNTHESIS |
| Enrichment Score (ES) | 0.39284748 |
| Normalized Enrichment Score (NES) | 1.2432548 |
| Nominal p-value | 0.16312057 |
| FDR q-value | 0.27326146 |
| FWER p-Value | 1.0 |
Table: GSEA Results Summary

  

Fig 1: Enrichment plot: KEGG\_O\_GLYCAN\_BIOSYNTHESIS      
 Profile of the Running ES Score & Positions of GeneSet Members on the Rank Ordered List

  

| SYMBOL | TITLE | RANK IN GENE LIST | RANK METRIC SCORE | RUNNING ES | CORE ENRICHMENT || 1 | C1GALT1C1 | C1GALT1 specific chaperone 1 [Source:HGNC Symbol;Acc:HGNC:24338] | 22 | 1.098 | 0.1626 | Yes |
| 2 | GALNT11 | polypeptide N-acetylgalactosaminyltransferase 11 [Source:HGNC Symbol;Acc:HGNC:19875] | 164 | 0.845 | 0.2782 | Yes |
| 3 | C1GALT1 | "core 1 synthase, glycoprotein-N-acetylgalactosamine 3-beta-galactosyltransferase 1 [Source:HGNC Symbol;Acc:HGNC:24337]" | 1110 | 0.494 | 0.2788 | Yes |
| 4 | GALNT10 | polypeptide N-acetylgalactosaminyltransferase 10 [Source:HGNC Symbol;Acc:HGNC:19873] | 1173 | 0.481 | 0.3461 | Yes |
| 5 | GCNT3 | "glucosaminyl (N-acetyl) transferase 3, mucin type [Source:HGNC Symbol;Acc:HGNC:4205]" | 1683 | 0.401 | 0.3666 | Yes |
| 6 | GCNT1 | glucosaminyl (N-acetyl) transferase 1 [Source:HGNC Symbol;Acc:HGNC:4203] | 2035 | 0.357 | 0.3928 | Yes |
| 7 | GALNT7 | polypeptide N-acetylgalactosaminyltransferase 7 [Source:HGNC Symbol;Acc:HGNC:4129] | 2954 | 0.255 | 0.3597 | No |
| 8 | GCNT4 | glucosaminyl (N-acetyl) transferase 4 [Source:HGNC Symbol;Acc:HGNC:17973] | 3858 | 0.169 | 0.3150 | No |
| 9 | GALNT4 | polypeptide N-acetylgalactosaminyltransferase 4 [Source:HGNC Symbol;Acc:HGNC:4126] | 5610 | 0.023 | 0.1825 | No |
| 10 | GALNT6 | polypeptide N-acetylgalactosaminyltransferase 6 [Source:HGNC Symbol;Acc:HGNC:4128] | 5915 | 0.001 | 0.1591 | No |
| 11 | GALNT1 | polypeptide N-acetylgalactosaminyltransferase 1 [Source:HGNC Symbol;Acc:HGNC:4123] | 6217 | -0.022 | 0.1390 | No |
| 12 | GALNT14 | polypeptide N-acetylgalactosaminyltransferase 14 [Source:HGNC Symbol;Acc:HGNC:22946] | 6762 | -0.060 | 0.1058 | No |
| 13 | GALNT2 | polypeptide N-acetylgalactosaminyltransferase 2 [Source:HGNC Symbol;Acc:HGNC:4124] | 7453 | -0.115 | 0.0694 | No |
| 14 | GALNT8 | polypeptide N-acetylgalactosaminyltransferase 8 [Source:HGNC Symbol;Acc:HGNC:4130] | 8124 | -0.164 | 0.0420 | No |
| 15 | B4GALT5 | "beta-1,4-galactosyltransferase 5 [Source:HGNC Symbol;Acc:HGNC:928]" | 9051 | -0.236 | 0.0054 | No |
| 16 | GALNT12 | polypeptide N-acetylgalactosaminyltransferase 12 [Source:HGNC Symbol;Acc:HGNC:19877] | 10414 | -0.362 | -0.0461 | No |
| 17 | GALNT3 | polypeptide N-acetylgalactosaminyltransferase 3 [Source:HGNC Symbol;Acc:HGNC:4125] | 11062 | -0.440 | -0.0304 | No |
| 18 | ST3GAL2 | "ST3 beta-galactoside alpha-2,3-sialyltransferase 2 [Source:HGNC Symbol;Acc:HGNC:10863]" | 11721 | -0.536 | -0.0012 | No |
| 19 | ST3GAL1 | "ST3 beta-galactoside alpha-2,3-sialyltransferase 1 [Source:HGNC Symbol;Acc:HGNC:10862]" | 12092 | -0.619 | 0.0627 | No |
Table: GSEA details [plain text format]

  

Fig 2: KEGG\_O\_GLYCAN\_BIOSYNTHESIS      
 Blue-Pink O' Gram in the Space of the Analyzed GeneSet

  

Fig 3: KEGG\_O\_GLYCAN\_BIOSYNTHESIS: Random ES distribution      
 Gene set null distribution of ES for **KEGG\_O\_GLYCAN\_BIOSYNTHESIS**
